# Wythe’s Pocket Dose and Symptom Book…. The Physician’s Visiting List, for 1862

**Published:** 1861-10

**Authors:** 


The same gentleman also sends us, for the publishers, Lindsay & Blakiston, Philadelphia, Wythe's *Pocket Dose and*

*Symptom Book*, and the *Physician's Visiting List*, for 1862. These little conveniences are so well and favorably known to the profession that we deem it only necessary to announce their publication. Wythe's work is in its third edition, and contains the doses and uses of every officinal remedy and preparation; nearly every useful native medical plant, and a variety of remedies recently introduced to the profession. The part devoted to symptoms of disease, and the outline of General Pathology and Therapeutics, will be found useful by the student. The classification of the Materia Medica, the table of weights and measures, add to the value of the work, and render it a *vade mecum*, in which the most useful information is compressed into the smallest possible compass, for the benefit of treacherous memories.

